# Arginine auxotrophic gene signature in paediatric sarcomas and brain tumours provides a viable target for arginine depletion therapies

**DOI:** 10.18632/oncotarget.18843

**Published:** 2017-06-29

**Authors:** Ashley Vardon, Madhumita Dandapani, Daryl Cheng, Paul Cheng, Carmela De Santo, Francis Mussai

**Affiliations:** ^1^ Institute of Immunology and Immunotherapy, University of Birmingham, Birmingham, United Kingdom; ^2^ Bio-Cancer Treatment International Ltd, Hong Kong, China

**Keywords:** paediatric, sarcomas, brain, arginine, auxotrophism

## Abstract

Paediatric sarcomas and brain tumours, remain cancers of significant unmet need, with a poor prognosis for patients with high risk disease or those who relapse, and significant morbidities from treatment for those that survive using standard treatment approaches. Novel treatment strategies, based on the underlying tumour biology, are needed to improve outcomes. Arginine is a semi-essential amino acid that is imported from the extracellular microenvironment or recycled from intracellular precursors through the combined expression of the enzymes ornithine transcarbamylase (OTC), argininosuccinate synthase (ASS) and argininosuccinate lyase (ASL) enzymes. The failure to express at least one of these recycling enzymes makes cells reliant on extracellular arginine – a state known as arginine auxotrophism. Here we show in large in silico patient cohorts that paediatric sarcomas and brain tumours express predominately the arginine transporter SLC7A1 and the arginine metabolising enzyme Arginase 2 (ARG2), but have low-absent expression of OTC. The arginine metabolic pathway correlated with the expression of genes associated with tumour pathogenesis, and overall survival in paediatric sarcomas. This gene signature of arginine auxotrophism indicates paediatric sarcomas and brain tumours are a viable target for therapeutic arginase drugs under current clinical trial development.

## INTRODUCTION

Brain tumours and sarcomas account for around 50% of all paediatric tumours. Despite significant improvements in survival for paediatric cancer, these two groups of tumours have significantly inferior outcomes when compared with leukaemias and solid tumours such as Wilms’ and germ cell tumours [1].

Paediatric sarcomas are composed primarily of 3 major subtypes – osteosarcoma, Ewing's sarcoma, and rhabdomyosarcomas, whilst brain tumours are classified based on site, cell of origin and grade of tumour [2, 3].

For both groups of patients improvements in outcomes over the past 30 years have been achieved through the use of multi-drug cytotoxic chemotherapy, in conjunction with local control through surgery and radiotherapy. For patients that are cured, the morbidities of treatment may be significant not least due to surgical excision of limbs or organ tissue, and the long-term effects of chemotherapy. In particular local control measures for CNS tumours cause significant late effects in growth, endocrine regulation, as well as potential visual and hearing loss and significant neurocognitive and developmental effects [4]. For patients who present with high-risk sarcoma such as alveolar rhabdomyosarcoma, metastatic osteosarcoma, or those who relapse the prognosis remains dismal. Similarly, certain types of CNS tumours, such as embryonal tumours (atypical teratoid rhabdoid tumours, pineoblastoma) and the high grade tumours such as grade III and IV gliomas and medulloblastomas with metastases have an extremely poor prognosis. Diffuse intrinsic pontine gliomas (DIPG) is universally fatal with median survival of 9 months from diagnosis [5].

For both groups of patients, there has been a paucity of new and effective therapeutic strategies, often secondary to the limitations of accessing tumour tissue and modelling the underlying tumour biology. Therefore, there is a strong case for investigating new pathways in these tumours. Recently, targeting tumour amino acid metabolism has become a clinically-relevant therapeutic approach [6]. Arginine is a semi-essential amino acid which plays a key role in protein synthesis, polyamine synthesis, and the cell cycle. Arginine is transported from the extracellular microenvironment via the SLC7A family of transporters and catabolised through the tissue-specific expression of Arginase 1 (ARG1), Arginase 2 (ARG2), or Nitric Oxide Synthetase (iNOS) enzymes. Arginine can also be recycled from the precursors ornithine and citrulline through the intracellular expression of ornithine transcarbamylase (OTC), argininosuccinate synthase (ASS) and argininosuccinate lyase (ASL) enzymes. The failure to express at least one of these recycling enzymes makes cells reliant on extracellular arginine – a state known as arginine auxotrophism. It is increasingly recognised that a number of adult malignancies are deficient in one of more of these arginine-recycling enzymes, making them susceptible to a new class of therapeutic arginine depleting enzymes undergoing clinical investigation. However to date the status of the arginine metabolic pathway has never been reported in paediatric sarcomas and brain tumours despite the unmet clinical need. Using large in silico data sets from human tumours, we demonstrate that paediatric sarcomas and brain tumour subtypes are arginine auxotrophic, supporting the translation of arginine-depletion strategies for these patients.

## RESULTS

Key components of arginine metabolism by cells include the ability to transport arginine intracellularly from the microenvironment, catabolise arginine into intermediates for downstream pathways, and the ability to re-synthesise arginine from precursor metabolites. We analysed the expression of the key arginine pathway genes in paediatric sarcomas (127 osteosarcoma samples, 117 Ewing's sarcoma samples, and 147 rhabdomyosarcoma samples) and paediatric CNS tumours (comprising 18 ATRT, 53 high grade glioma, 37 DIPG and 83 ependymoma, 73 medulloblastoma and 182 CNS-PNET) held within the R2: genomics analysis and visualization platform. This represents the largest analysis of paediatric tumours from patients, for arginine pathway alterations described in the literature. Data was revalidated against at least one other independent data set of the same tumour type (Supplementary Figure 1), confirming the same expression profiles.

### Expression of arginine pathway genes in paediatric sarcomas and brain tumours

**Figure 1:**
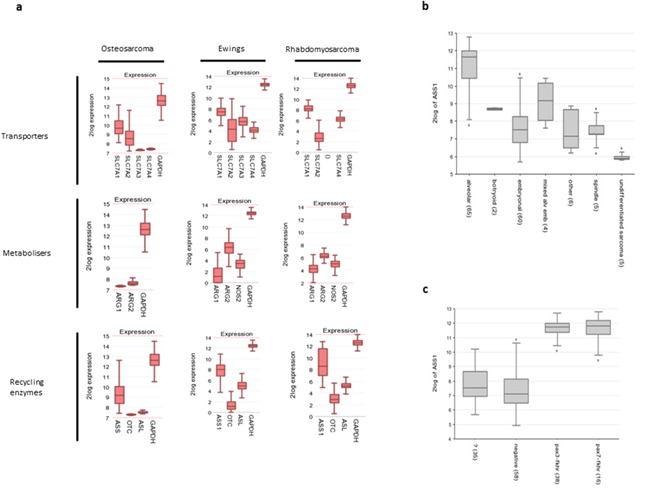
Paediatric sarcomas have an arginine auxotrophic gene signature **(a)** Gene expression profiles of amino acid transporters, arginine catabolic enzymes, and arginine recycling enzymes, compared to GAPDH control. **(b)** ASS expression in rhabdomyosarcoma histological subgroups **(c)** ASS expression according to rhabdomyosarcoma cytogenetic status.

Within osteosarcoma (Figure 1a) we identified that cells have higher expression of the SLC7A1 and SLC7A2 amino acid transporters, compared to the SLC7A3 and SLC7A4 transporters (Figure 1a) consistent with the established role of these isoforms in transporting arginine and other key amino acids such as lysine and ornithine. In osteosarcoma, both ARG1 and ARG2 catabolic enzymes were moderately expressed. No data available for NOS2. Although osteosarcomas expressed ASS, expression of OTC and ASL were lower in comparison. Enzyme expression did not correlate with clinical features of the patient, histological subtype, anatomical location of the primary tumour, or response to chemotherapy.

For Ewing's sarcoma (Figure 1a) the expression levels of amino acid transporters are more equally distributed although SLC7A1 was again the most predominant. ARG2 expression was the main isoform expressed with low-absent ARG1, and low levels of NOS2. Of the arginine recycling enzymes, OTC expression was low-absent, with higher expression of ASS1 and ASL. Expression of these enzymes did not correlate with clinical features.

In rhabdomyosarcomas (Figure 1a) SLC7A1 and SLC7A4 were strongly expressed, with low SLC7A2 (SLC7A3 not available). Similar to the other sarcomas expression of ARG2 was highest, with modest expression of ARG1 and NOS2 genes. OTC expression was again significantly lower than ASS1, with comparable expression of ASL. Between histological subtypes of tumour ASS expression was significantly different (p=3.6e-19, ANOVA), and highest in the patient group with the worst prognosis - alveolar rhabdomyosarcoma (Figure 1b). ASS1 expression also corresponded with PAX3/PAX7-FKHR expression (p=4.6e-46), consistent with this finding (Figure 1c).

These findings show that these major paediatric sarcoma subtypes have the ability to transport arginine from the microenvironment and metabolise it. However, despite relatively preserved ASS and ASL expression they have a low or absent OTC indicating the potential of arginine auxotrophism.

**Figure 2:**
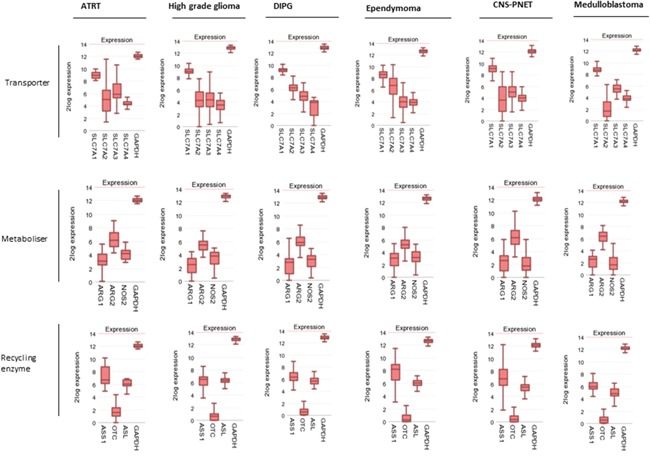
Paediatric brain tumours have an arginine auxotrophic gene signature Gene expression profiles of amino acid transporters, arginine catabolic enzymes, and arginine recycling enzymes compared to. GAPDH control.

Within CNS tumours the expression pattern of arginine pathway genes was consistent across all subtypes (Figure 2). SLC7A1 was the highest expressed transporter isoform with ARG2 as the main catabolic enzyme. OTC expression was very low or completely absent throughout all tumour subtypes with relatively preserved ASL and ASS expression. Thus all CNS tumour types share a predisposition to arginine auxotrophism.

### Impact of arginine pathway genes on overall survival

**Figure 3:**
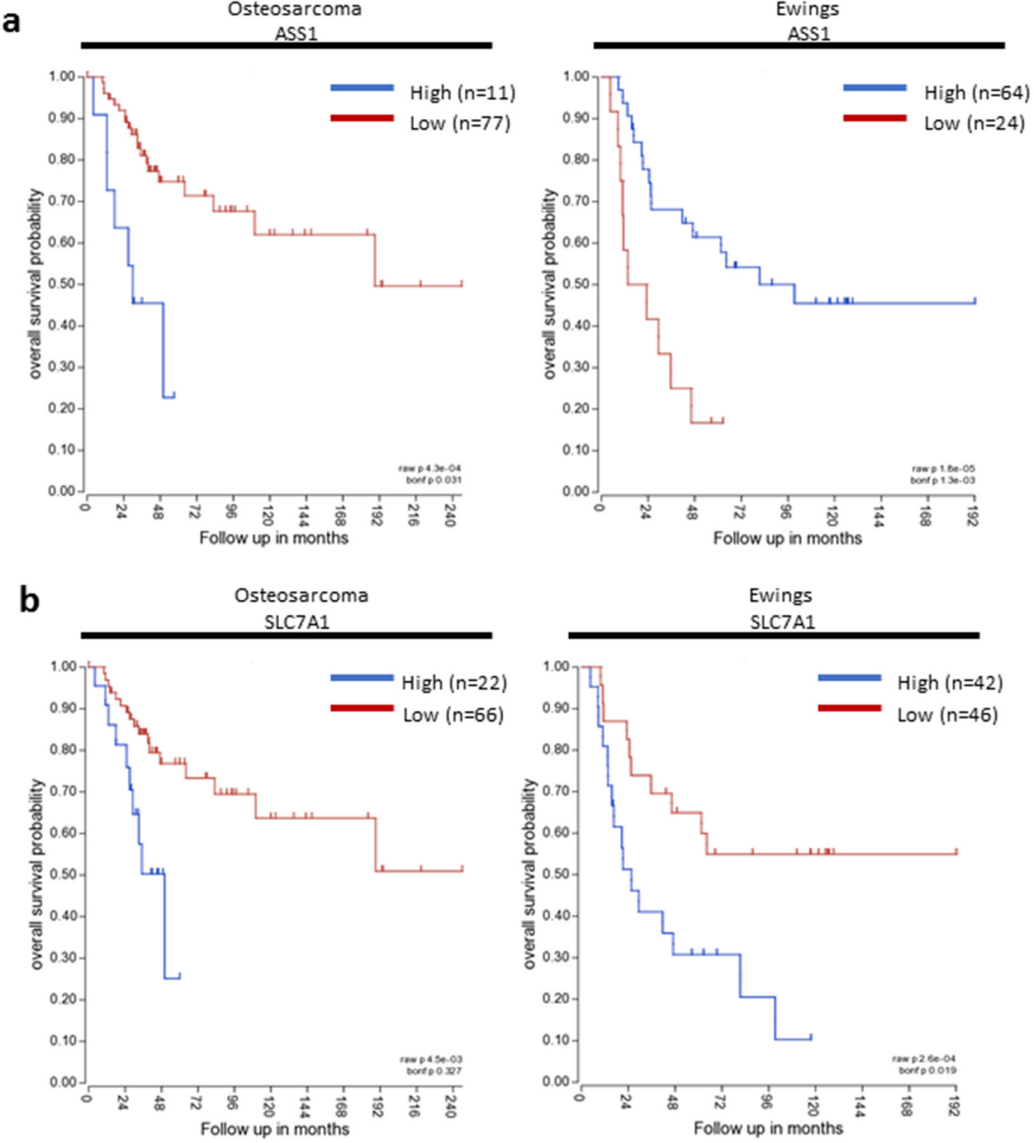
SLC7A1 and ASS expression correlate with survival in paediatric sarcomas Kaplan-Meier survival curves based on SLC7A1 and ASS expression in osteosarcoma and Ewing's sarcoma. Blue – High gene expression. Red- Low gene expression.

The analysis above indicates that the major paediatric sarcomas universally express SLC7A1, ARG2, and ASS, but have low OTC expression. We therefore assessed the impact of arginine pathway genes on overall survival in osteosarcoma and Ewing's sarcoma patients (Figure 3). Data on rhabdomyosarcoma was not available.

In osteosarcoma, high ASS expression was associated with poorer overall survival probability (p=0.031). No significant difference in overall survival was detected based on expression of SLC7A1, ARG2 or OTC. In contrast for Ewing's sarcoma, low ASS expression was associated with poorer overall survival (p=0.0013), and high SLC7A1 expression was associated with poorer overall survival (p=0.019). Expression of ARG2 and OTC did not significantly correlate with overall survival. Differences in relative gene expression on survival for each tumour, is an indicator of the underlying varied tumour biology and the impact of other interacting cellular pathways.

Survival data was limited in the CNS tumours examined. Only survival data for high grade glioma was available of all the datasets used. There was no significant difference in overall survival in high grade glioma with SLC7A1, ARG2, ASS or OTC expression.

### The arginine auxotrophic signature correlates with other intracellular targets for novel agents

Gene set enrichment was carried out for all sarcoma and CNS tumour subtypes, using pre-ranked gene lists correlating with ARG2 and OTC (Supplementary Figure 2). In all tumour types studied, except glioma, ARG2 and OTC ranked gene lists significantly correlated with KRAS signaling. MTOR signaling, a known downstream effector of amino acid metabolism and a target for novel agents in relapsed paediatric tumours, was enriched within osteosarcoma for both OTC and ARG2 (Supplementary Figure 2a). In medulloblastoma MYC (associated with a poor prognosis) had a positive correlation with FDR <0.05, for both the OTC and ARG2 oncogenic and hallmark gene lists (Supplementary Figure 2i). In ependymoma EGFR upregulation, a target of monoclonal antibodies and small molecule inhibitors, correlated with ARG2 (FDR 0.000) (Supplementary Figure 2g). ERB2 upregulation, targeted recently with small molecule inhibitors, also correlated with the OTC ranked gene list (FDR 0.000) for DIPG (Supplementary Figure 2f).

The interplay of the immune microenvironment and arginine metabolism as a contributor to tumour pathogenesis was also highlighted from our analysis. The continuing growth of immunotherapeutics makes targeting these pathways with anti-cytokine monoclonal antibodies of interest. In ependymomas and DIPG IL-6-JAK/STAT, IFN-γ, and TNF-α signaling were enriched (Supplementary Figure 2f and 2g). The NF-KB complex, a key player in immune and inflammatory signaling, was significantly enriched in both the hallmark and oncogenic data sets for gliomas, ependymomas, ATRT and Ewing's sarcoma. The above findings show that in both paediatric sarcoma and brain tumours, arginine metabolism influences a number of key pathways in cell and tumour development, and could stimulate novel therapeutic approaches in combination with arginine depletion therapy.

## DISCUSSION

Cancer cell arginine auxotrophism is determined by 3 core cellular components: the ability to transport arginine from the extracellular microenvironment into the cell; the expression of arginine catabolising enzymes; and the absence of arginine recycling pathway enzymes [6]. Arginine is transported from the extracellular microenvironment into the cell cytoplasm predominantly through the system+ family of cationic amino acid transporters (SLC7A1, SLC7A2, SLC7A3, SLC7A4). 3 enzymes are principally involved in the catabolism of arginine. Arginase 1 (ARG1) and arginase 2 (ARG2) catabolise arginine in ornithine and urea, whilst nitric oxide synthetase (NOS2) converts arginine into citrulline and nitric oxide. In the majority of non-malignant cells arginine breakdown products are recycled back to arginine, through the expression of Ornithine transcarbamylase (OTC; converting ornithine into citrulline), Argininosuccinate Synthetase (ASS; converting citrulline into argininosuccinate), and finally Argininosuccinate Lyase (ASL; converting argininosuccinate back to arginine) (Supplementary Figure 3).

Here we identify, in large cohorts of paediatric tumours, that the arginine transporter SLC7A1 is highly expressed. ARG2 was the main catabolic enzyme found within the tissues, with lower expression of ARGI and iNOS enzymes. Although ASS was universally expressed, OTC was significantly underexpressed in all three subgroups of sarcomas and absent of very low in all CNS tumours. To date the status of the arginine metabolic pathway in human samples from paediatric sarcomas and brain tumours has never been reported.

The SLC7A family of transporters have been described in both non-malignant and adult malignant cells as the principle method of cellular arginine uptake from the microenvironment. We have shown that SLC7A1 is predominant in both adult and paediatric Acute Myeloid Leukaemia blasts which are dependent on arginine for survival [25]. Modulation of SLC7A1 expression in colorectal carcinoma and breast cancer cells decreases arginine uptake and reduces their proliferation and survival [26]. As SLC7A1 has an upstream open reading frame (uOR) in its proximal promoter which can be translated under conditions of arginine depletion, it suggests that this transporter is the cellular response to arginine concentrations in the microenvironment [27, 28]. Although the expression of other SLC7a family members has been reported to play a role in cancer associated myeloid-derived suppressor cells and in non-small cell lung cancer radiation resistance little is known about the unique functions of each SCL7A family member in the cancer pathogenesis, in particular in the paediatric setting [29, 30].

The paediatric tumours examined here show a predominance for Arginase II expression, over Arginase I and INOS. Both Arginase I and II catalyse the conversion of arginine into urea and ornithine, although they are reported to be localised in the cytoplasm and mitochondria respectively. The over-expression of arginase enzymes has been reported in adult soft tissue sarcomas with no correlation to histopathological parameters [31]. Hypoxia can drive Arginase II dependent proliferation of Osteosarcoma cell lines [32]. However the expression and function of Arginase enzymes in the malignant cells of human paediatric sarcomas and brain tumours has never been described to date. Thus the specific contribution of each of these enzymes in promoting tumour cell proliferation is still unclear although it is well established that a number of downstream pathways are dependent on arginine metabolism – such as mTOR regulation, ATF2 dependent regulation of cell cycle in leukaemia, and polyamine synthesis in neuroblastoma [33–55]. We have previously shown that Arginase II, is the main arginine catabolic enzyme in AML blasts and neuroblastoma tumour cells, and that its expression contributes to the creation of an immunosuppressive microenvironment [36, 37]. Indeed these tumour cells were found to be low or absent in Arginase I or iNOS enzymes. In contrast we have shown that cancer-induced myeloid-derived suppressor cells (MDSCs) can overexpress Arginase I and iNOS enzymes, to impair T cell proliferation and activation [38, 39]. Murine models of paediatric sarcomas, gliomas, and glioblastomas have been shown to induce Arginase 1+/iNOS+ MDSCs [40–43]. Our data supports that the tumour microenvironment in paediatric sarcomas and brain tumours is likely to be arginine deplete, and thus a suppressive environment for anti-cancer immunity.

The central tenant of arginine auxotrophy is that tumour cells have an incomplete complement of arginine resynthesis enzymes. Ornithine, produced by arginine breakdown, is converted first into citrulline through a reaction with carbamoyl phosphate by OTC. Here we show that paediatric sarcomas and brain tumours have low to absent expression of this enzyme, thus breaking the recycling pathway. In AML we showed that OTC has a range of expression in both adult and paediatric blasts. However in a range of adult solid cancer cell lines (keratinoctic carcinoma, lung adenocarcinoma, hepatocellular carcinoma and breast carcinoma) OTC was undetectable consistent with our findings here [44, 45]. An absence of OTC may suggest that tumours use ornithine for polyamine synthesis and not arginine regenertation [46]. Our data supports previous findings that no ASL negative tumours have been identified. The function of ASL can be regulated by fumarate hydratase expression in renal cell carcinoma and by epigenetic modulation in glioblastoma [47].

A number of genes correlated with the expression of arginase enzymes in paediatric tumours, in particular related to transcription factors and intracellular signal transduction. Although no single pathway was prevalent, corresponding with the different underlying biology of these tumours, these findings do suggest arginine metabolism has a wide influence on cellular functions. The most significant signalling hallmarks and genes enriched in the gene set enrichment analysis for the paeditatric tumours analysed here, was KRAS and KRAS signalling pathway. The pathway plays an established role in cell proliferation and differentiation. A number of human genetic syndromes have emerged that are caused by germline mutations in components of the Ras/mitogen activated protein kinase (MAPK) pathway, and are associated with an increased risk of developing cancer [48]. Transgenic expression of KRAS in neural stem and progenitor cells induces brain tumorigenesis in a zebra fish model [49]. Dysregulated KRAS signaling has also been implicated in rhabdomyosarcoma and Ewing's sarcoma pathogenesis [50, 51].

As such targeting arginine metabolism could provide a new therapeutic approach for these childhood cancers of significant unmet need. Until recently, drugs targeting arginine metabolism in a clinically relevant manner did not exist. Small molecules which inhibit arginine transport or arginase enzymes are only available for *in vitro* research use (N^G^-hydroxy-L-arginine: NOHA and L-N^G^-monomethyl arginine: L-NMMA) and suffer from a number of issues. Inhibitors of SLC7 transporters are likely to lead to toxicity from inhibiting uptake of other amino acids by non-malignant tissues as well as the tumour cells, and due to the structural homology between different SLC7 family members. The arginase I and iNOS enzyme inhibitors are commonly arginine analogues which are reversible in binding to the enzymes, and due to the close interplay between iNOS and Arginase pathways require concurrent administration in order to fully inhibit cellular use of arginine. New arginase I inhibitors, such as CB-1158 (Calithera, Inc) are at various stages of preclinical and early clinical development (NCT02903914).

The most clinical relevant approach to targeting tumour arginine metabolism is through therapeutic arginine depletion. Normal human arginase has limited clinical value because of a short plasma-half life. We have shown that the addition of a 5000 MW polyethylene glycol molecule (PEG) significantly increases the plasma half-life of arginase I with minimal loss of enzyme activity. BCT-100 is a pegylated recombinant human arginase which we have demonstrated to have significant pre-clinical activity against leukaemias, hepatocellular carcinoma, melanoma, and prostate cancer [52, 53]. Phase I and II clinical trials have been completed in adults with relapsed/refractory hepatocellular carcinoma, demonstrating that BCT-100 can lead to a sustained depletion of plasma arginine to undetectable levels (<8uM) for 5 days or more with weekly dosing [54, 55]. Patients experienced a significant improvement in overall survival with an excellent toxicity profile. Pegyated mycoplasma derived arginine demininase (PEG-ADI) is an alternative strategy that catabolises arginine into citrulline and ammonia [56]. The immunogenicity of the molecule has significantly limited sustained arginine depletion and anti-tumour efficacy of this molecule.

Our findings suggest future clinical studies could rationally include a combination of arginase therapy in combination with molecules targeting other intersecting intracellular pathways. A number of early phase clinical trials are ongoing in the field of relapsed paediatric malignancies, including those targeting the ERBB2, EGFR, MTOR, and Aurora Kinase pathways, we identified as correlating with arginine metabolism [57–59]. Sarcomas remain a disease of significant unmet clinical need, with a lack of new agents in upfront or relapsed treatment protocols. *In vitro* cell line studies have shown that paediatric sarcomas including osteosarcoma, Ewing's sarcoma, synovial sarcoma and malignant peripheral nerves sheath tumours, overexpress arginine metabolising enzymes consistent with a dependence on arginine for growth. Resistance to doxorubicin, one of the principal chemotherapy agents used in sarcoma treatment, is associated with ASS1 downregulation, suggesting relapsed sarcomas are more likely to be arginine auxotrophic [60]. Immunohistochemical analysis of sarcomas shows they may have reduced or absent ASS1 expression. Arginine deprivation induces cell death of osteosarcoma, Ewing's sarcoma, and synovial sarcoma cell lines by cell cycle arrest and subsequent autophagy or necroptosis *in vitro*, and inhibition of tumour growth in murine xenografts [61, 62]. For High Grade Gliomas disease representative preclinical models are still in early stages of development, contributing to a paucity of new agents for this group of patients. The immediate downstream metabolite of arginine - polyamine, plays a critical role in medulloblastoma and glioblastoma cell pathogenesis [63, 64]. Glioblastoma cells have been shown to undergo alterations in invasiveness in low arginine conditions [65]. Cell lines undergo caspase-independent cell death *in vitro* and have a significant reduction in in xenograft growth when treated with arginine depleting enzymes [66, 67].

Our study is limited to the patient cohorts within the R2: Genomics Analysis and Visualization platform and generates a number of hypotheses of the role of arginine in tumour cells. Although this provides data on large datasets from primary patient samples, further *in vitro* and *in vivo* studies is necessary to further investigate how arginine metabolism can be functionally linked to other biological pathways and the impact of therapeutic approaches. Overall this study highlights an arginine auxotrophic signature in paediatric sarcomas and brain tumours and provides an important rationale for investigating clinically relevant arginine depletion strategies in these children.

## MATERIALS AND METHODS

### Arginine pathway gene expression profiles

Using the R2: Genomics Analysis and Visualization Platform (http://r2.amc.nl), we analysed the expression of argininosuccinate synthetase 1 (ASS1); ornithine transcarbamylase (OTC); argininosuccinate lyase (ASL), nitric oxide synthase 2 (NOS2), Arginase 1 and 2 (ARG1 and ARG2) isoforms and the solute carrier family/cationic amino acid transporters SLC7A1, SLC7A2, SLC7A3, and SLC7A4, and the housekeeping gene GAPDH in the range of published sarcoma and CNS tumour datasets as described in Table 1 [8–24]. Arginine auxotrophy was defined as the low or absent expression of ASS alone or in combination with low or absent expression of OTC. The relative expression of the genes are shown, including GAPDH and TUBB as housekeeping genes.

**Table 1:** Table of patient cohorts analysed

Tumour type	Database name	Data features and Source Pubmed reference
**DIPG**	Glioma DIPG - Paugh - 37 - MAS5.0 - u133p2	n= 43 Affymetrix Human Genome U133 Plus 2.0 arrays. https://www.ncbi.nlm.nih.gov/pmc/articles/PMC3209696/ [8]
**Glioma**	Glioma - French - 284 - MAS5.0 - u133p2 Glioma pediatric - Paugh - 53 - MAS5.0 - u133p2	n=28 Affymetrix https://www.ncbi.nlm.nih.gov/pubmed/19920198 [9] n=53 Affymetrix Human Genome U133 Plus 2.0 arrays https://www.ncbi.nlm.nih.gov/pubmed?term=20479398 [10]
**ATRT**	ATRT - Kool - 49 - MAS5.0 - u133p2 ATRT - Birks - 18 - MAS5.0 - u133p2	n=49 Affymetrix GeneChip Human Genome U133. https://www.ncbi.nlm.nih.gov/pubmed/26923874 [11] n=18 Affymetrix HG-U133 Plus 2 GeneChip microarrays. https://www.ncbi.nlm.nih.gov/pubmed?term=21946044 [12]
**Ependymoma**	Ependymoma - Donson - 19 - MAS5.0 - u133p2 Ependymoma - Gilbertson - 83 - MAS5.0 - u133p2	n=19 Affymetrix HG-U133 Plus 2 microarray chips https://www.ncbi.nlm.nih.gov/pubmed?term=19917695 [13] n=83 Affymetrix U133 Plusv2 and Agilent miRNA arrays https://www.ncbi.nlm.nih.gov/pubmed?term=20639864 [14]
**PNET**	CNS-PNET - Kool - 182 - MAS5.0 - u133p2 CNS/PNET - Grundy - 24 - MAS5.0 - u133p2	n=182 Affymetrix GeneChip Human Genome U133 Plus 2.0 Array. https://www.ncbi.nlm.nih.gov/pmc/articles/PMC5139621/ [15] n=24 Affymetrix U133 plus 2.0 arrays. https://www.ncbi.nlm.nih.gov/pubmed/21798848 [16]
**Medulloblastoma**	Medulloblastoma - Thompson - 46 - MAS5.0 - u133a Medulloblastoma PLoS One - Kool - 62 - MAS5.0 - u133p2	n=46 U133av2 Affymetrix oligonucleotide array. https://www.ncbi.nlm.nih.gov/pubmed/16567768 [17] n=62 Affymetrix HG-U133 plus 2.0. https://www.ncbi.nlm.nih.gov/pubmed?term=18769486 [18]
**Osteosarcoma**	Osteosarcoma - Kobayashi - 27 - MAS5.0 - u133p2 Osteosarcoma - Buddingh - 53 - vst - ilmnhwg6v2 Osteosarcoma - Kuijjer - 127 - vst - ilmnhwg6v2	n=27 Affymetrix Human Genome U133 Plus 2.0 array http://www.ncbi.nlm.nih.gov/pubmed?term=20159990 [19] n=53 Illumina Human-6 v2.0 Expression BeadChips http://www.ncbi.nlm.nih.gov/pubmed?term=21372215 [20] n=84 Illumina Human-6 v2.0 arrays/ http://www.ncbi.nlm.nih.gov/pubmed?term=22454324 [21]
**Ewings sarcoma**	Ewing Sarcoma - Delattre - 117 - MAS5.0 - u133p2 Ewing Sarcoma - Francesconi - 37 - MAS5.0 - u133p2	n=117 Affymetrix hgu133Plus2 arrays. http://www.ncbi.nlm.nih.gov/pubmed?term=22327514 [22] n=37 Affymetrix https://www.ncbi.nlm.nih.gov/pubmed/19307502 [23]
**Rhabdomyosarcoma**	Rhabdomyosarcoma - Davicioni - 147 - MAS5.0 - u133a	n=147 Affymetrix GeneChip Human U133A http://www.ncbi.nlm.nih.gov/pubmed?term=16849537 [24]

Databases used in the analysis are included in Table 1. To ensure there was no selection bias we validated findings using other databases with the same tumour type in the R2 visualisation platform. According the primary literature for each study Affymetrix and Illumina technologies were used and sample library preparation, hybridization, and quality control were performed according to manufacturer's protocol.

### Kaplan-Meier survival plots

Kaplan-Meier overall survival plots were generated using the R2: Kaplan Meier tool for the key SLC7A1, OTC, ASS and ARG2 genes, where data was available. Bonferroni corrected p-values were used.

### Gene set enrichment analysis

Gene set enrichment analysis was carried out, using ranked gene lists generated from the R2 platform (Table 1). Genes correlating with ARG2 and OTC were retrieved and ranked according to the correlation p-coefficient., An initial threshold of p<2 was used to generate the ranked genes list before enrichment analysis was carried out using Broad Institute software (http://software.broadinstitute.org/gsea/index.jsp). We used datasets from MsigDB (http://software.broadinstitute.org/gsea/msigdb/index.jsp) including ‘hallmark’ and ‘oncogenic signatures’. Oncogenic signatures were generated from microarray gene expression data from cancer gene perturbations. Hallmark gene sets were coherently expressed signatures derived by aggregating many MSigDB gene sets to represent well-defined biological states or processes. We used 1000 permutations with weighted enrichment statistics in the analysis. Hallmarks and oncogenic genes were selected on the basis of the 5 most significant or those with an FDR q value less than 25% (0.25).

## SUPPLEMENTARY MATERIALS FIGURES



## Abbreviations

ARG1: arginase 1
ARG2: arginase 2
ASL: argininosuccinate lyase
ASS: argininosuccinate synthase
DIPG: diffuse intrinsic pontine gliomas
MDSC: myeloid-derived suppressor cells
iNOS: nitric oxide synthetase
OTC: ornithine transcarbamylase
