# Supplementary material for: Arginine auxotrophic gene signature in paediatric sarcomas and brain tumours provides a viable target for arginine depletion therapies
Source: Oncotarget. 2017 Jun 29;8(38):63506–17. doi: 10.18632/oncotarget.18843 (PMC5609939; doi:10.18632/oncotarget.18843)
Supplement: Supplementary file 1 [file oncotarget-08-63506-s001.pdf]

Arginine auxotrophic gene signature in paediatric sarcomas and brain tumours provides a viable target for arginine depletion therapies

SUPPLEMENTARY MATERIALS

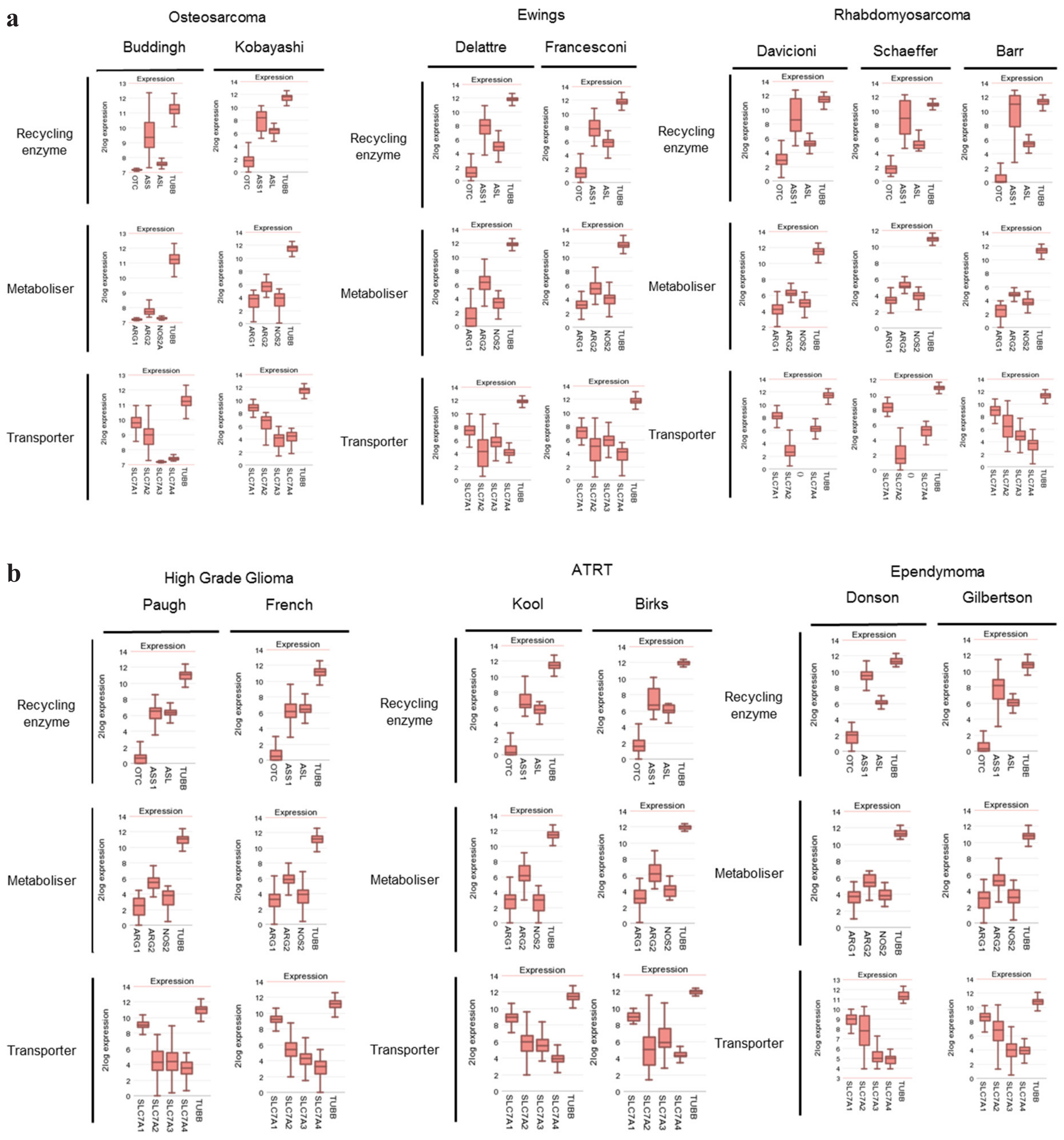

(Continued)

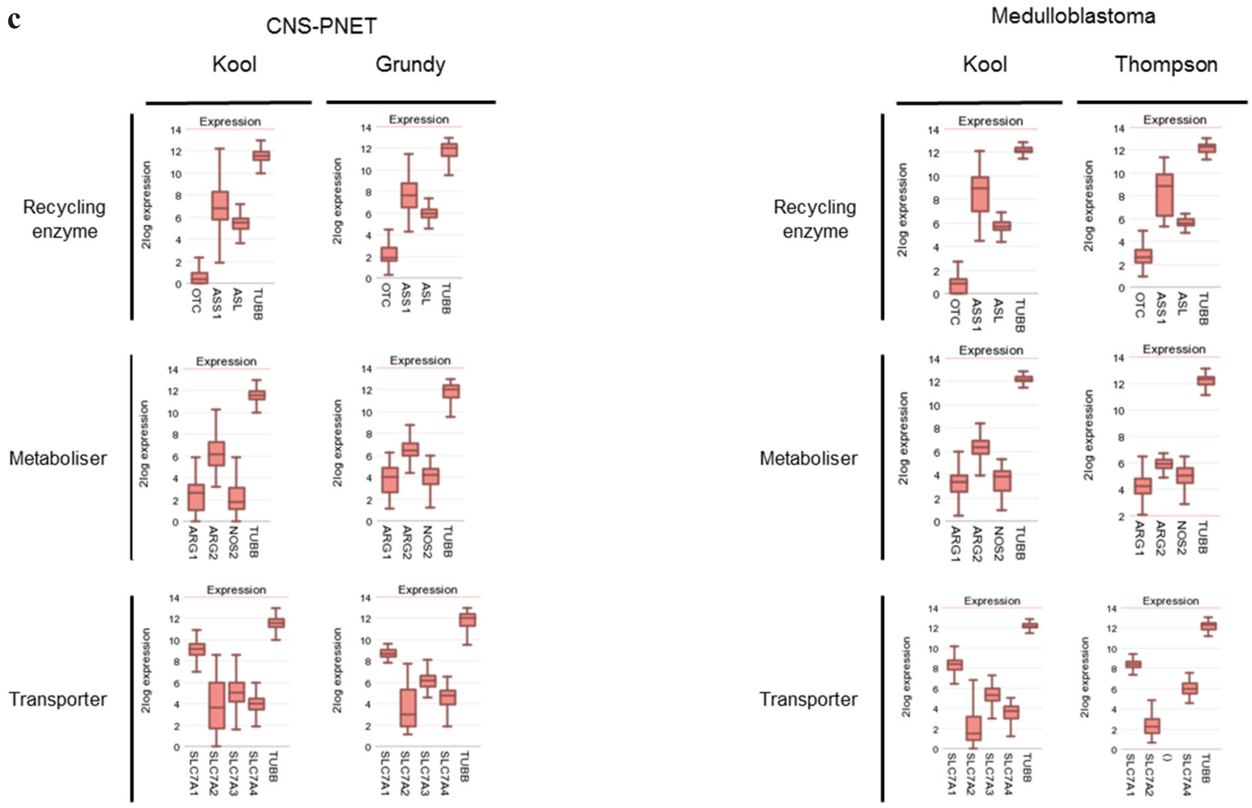

Supplementary Figure 1 (*Continued*): Validation analysis showing arginine auxotrophic gene signature in paediatric tumours. Gene expression profiles of amino acid transporters, arginine catabolic enzymes, and arginine recycling enzymes, compared to Tubulin control for **(a)** sarcomas, and brain tumours **(b** and **c)**.

a

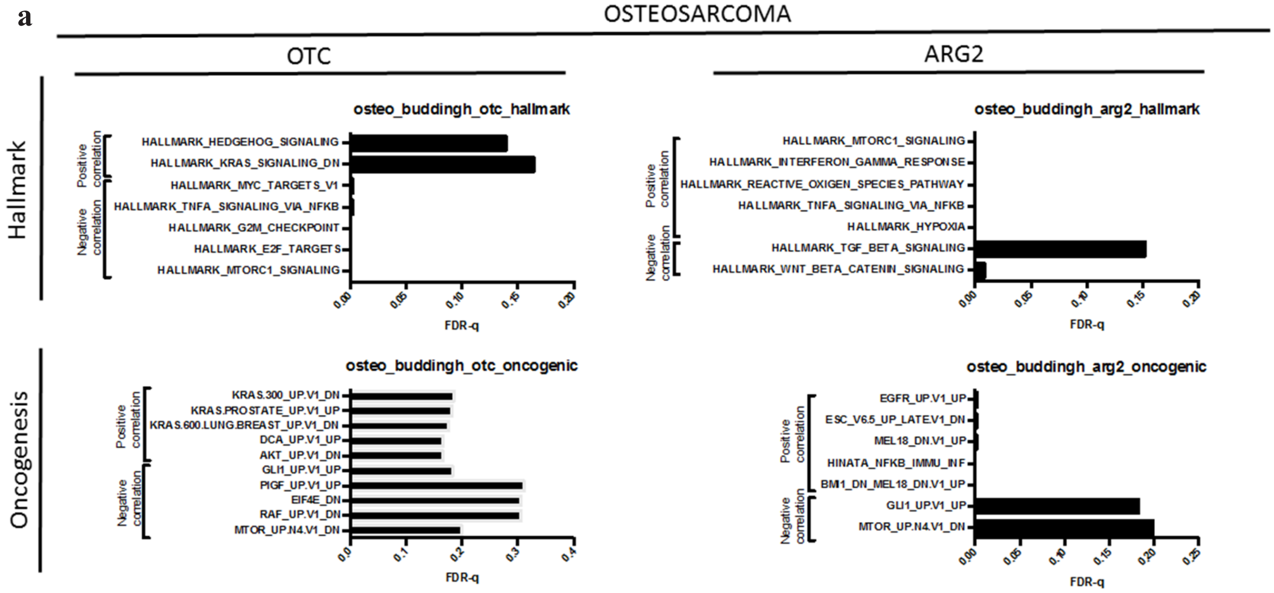

b

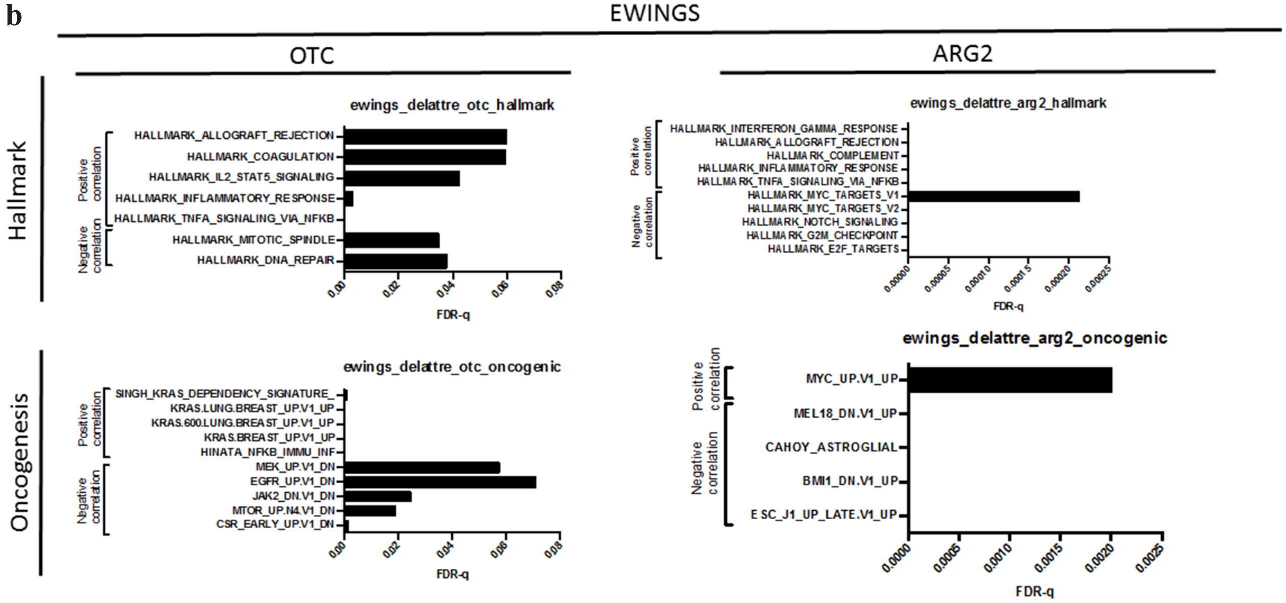

(*Continued*)

c

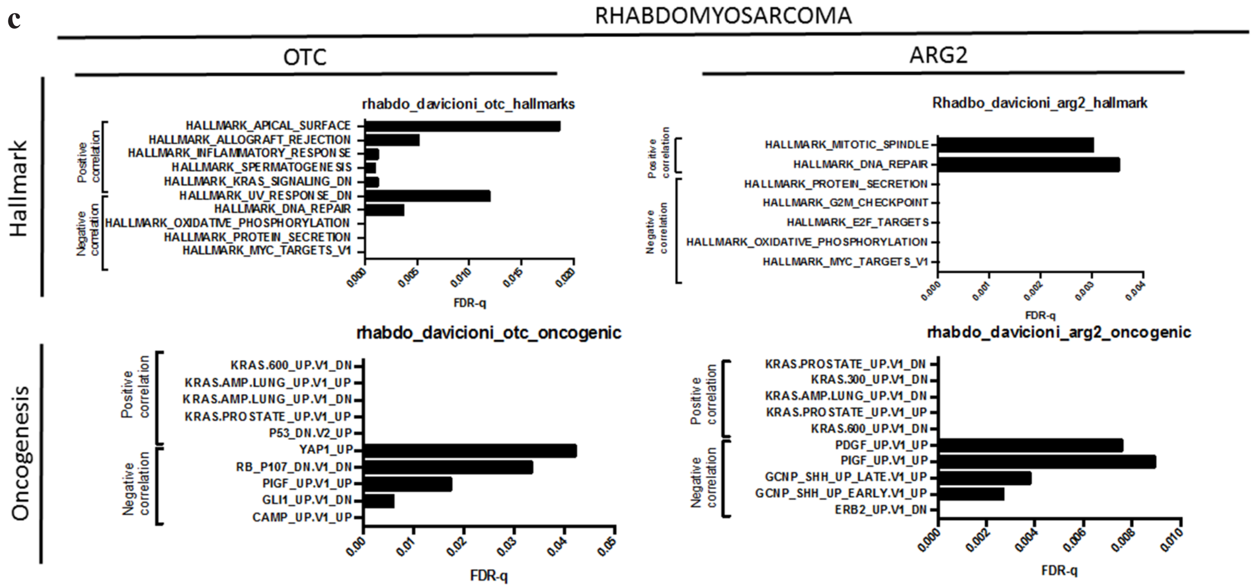

d

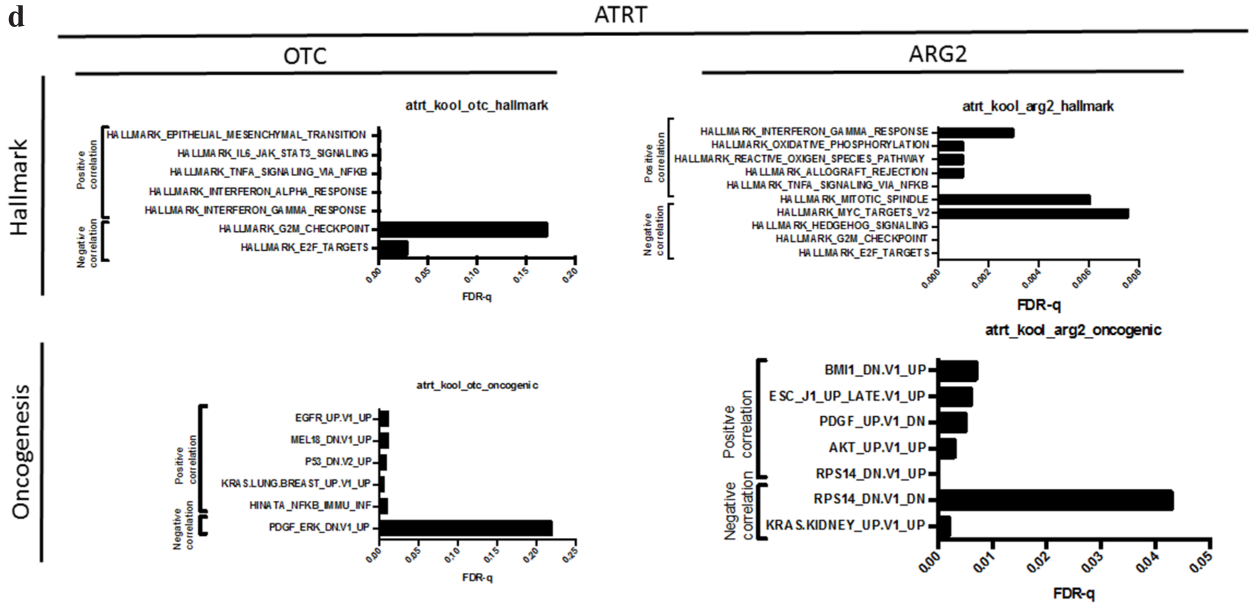

(*Continued*)

e

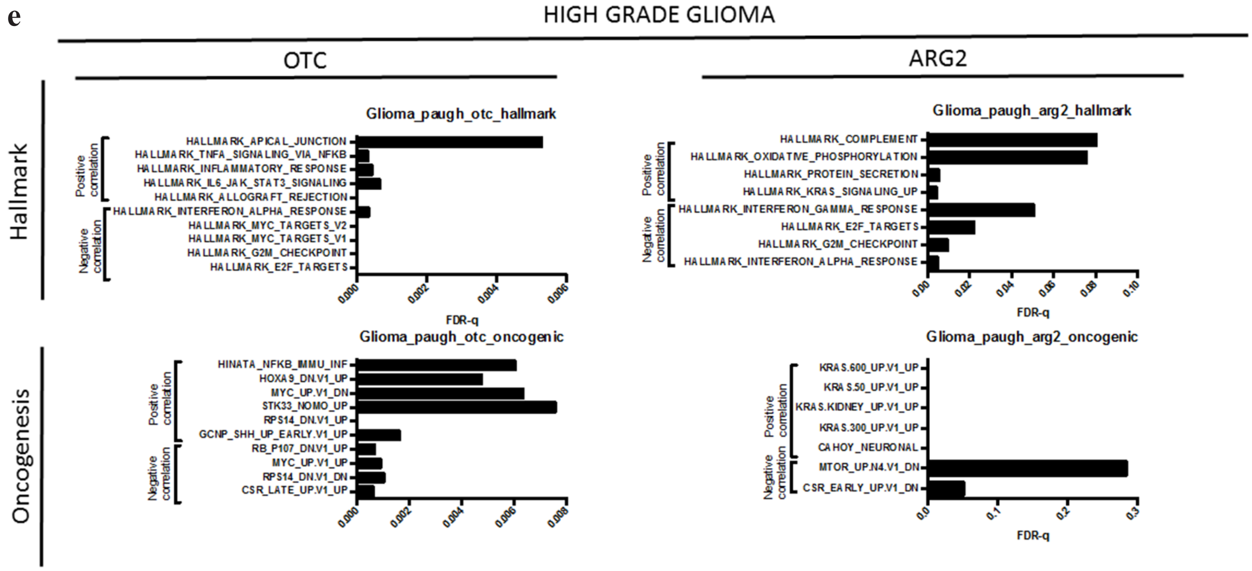

f

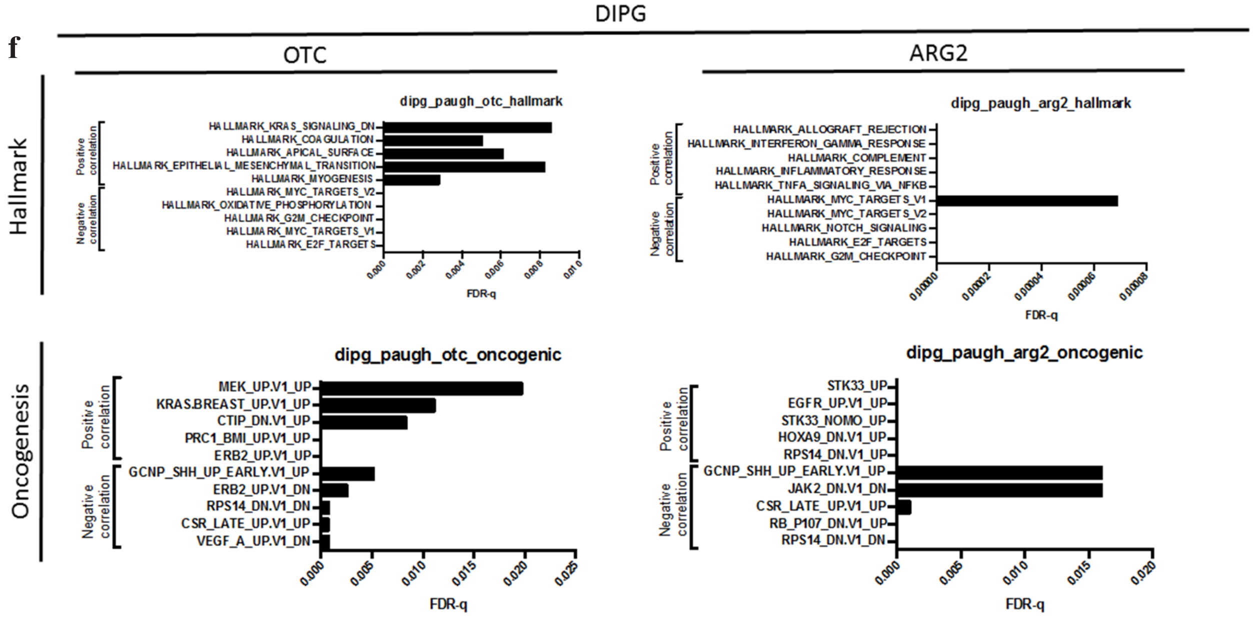

(Continued)

g

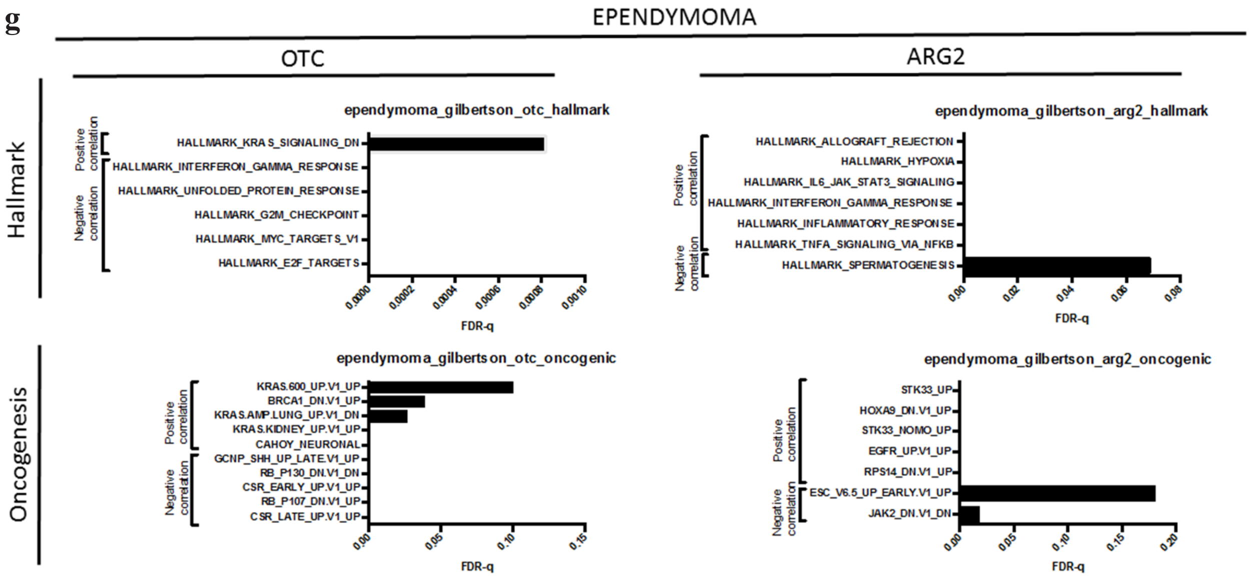

h

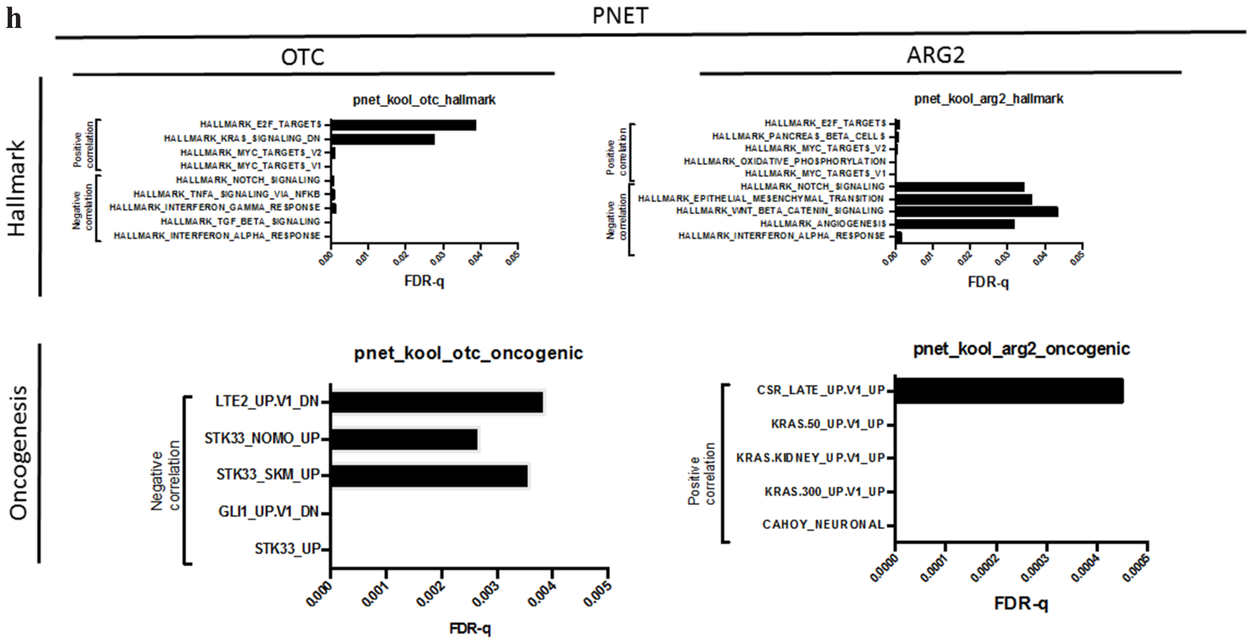

(Continued)

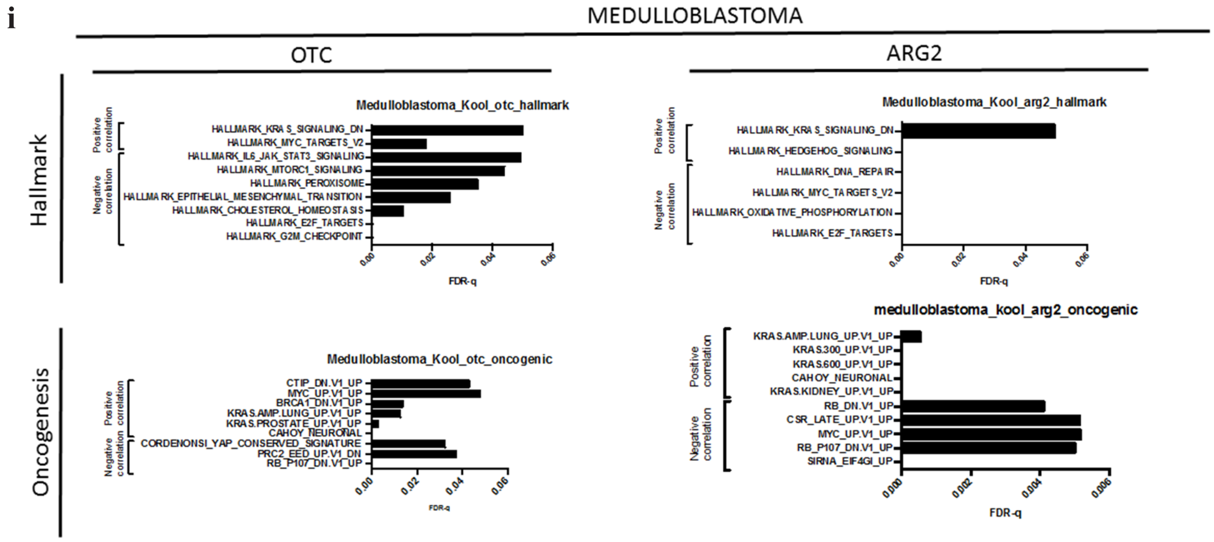

Supplementary Figure 2 (*Continued*): GESA analysis. GSEA figures showing hallmark or oncogenic signatures for OTC and ARG2, in each tumour type **(a-i)**. FDR-q values plotted. 0.00 represents a highly significant correlation.

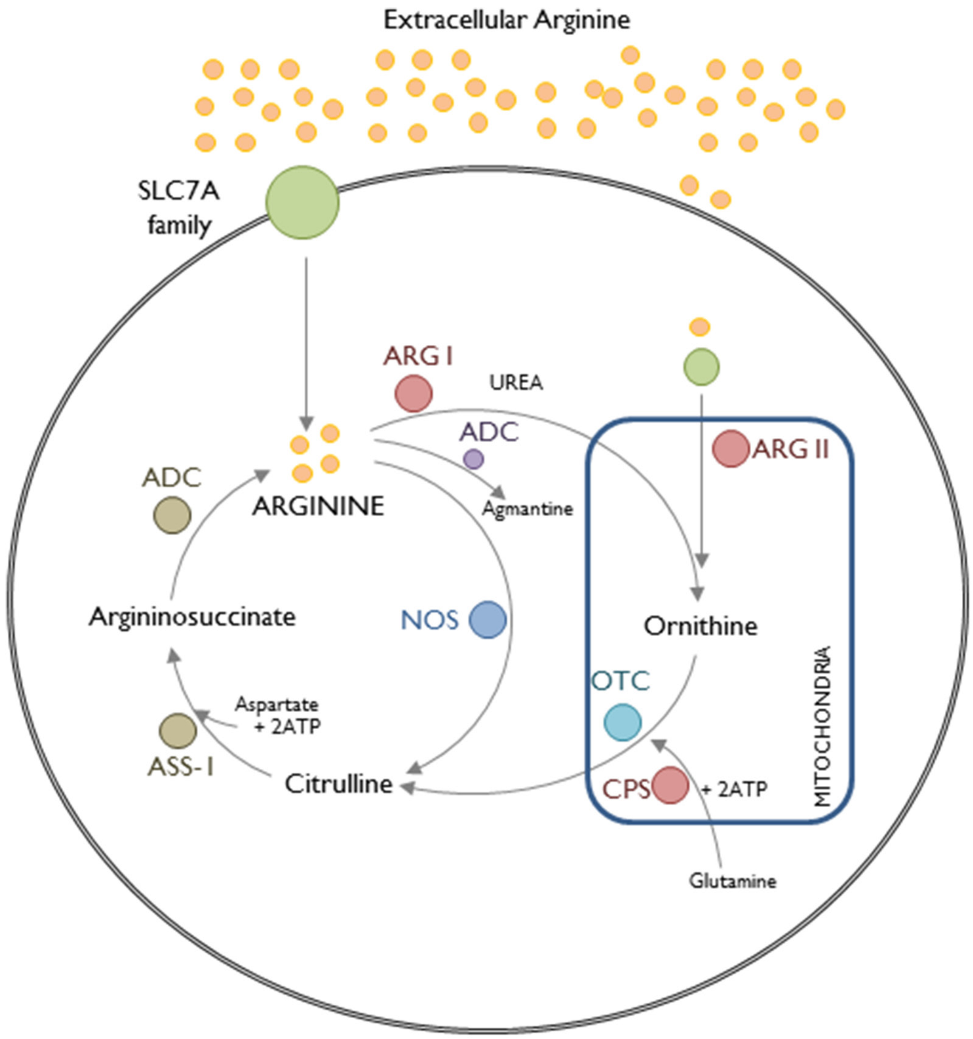

Supplementary Figure 3: Arginine metabolic pathway in cancer cells. Arginine is imported via the SLC7A family of membrane transporters. Intracellular arginine is metabolized by the enzymes Arginase I (ARGI) in the cytoplasm and/or Arginase II (ARGII) in the mitochondria, converting arginine to urea and ornithine. Ornithine transcarbamylase (OTC) converts ornithine to citrulline. Additional citrulline is produced via the activity of nitric oxide synthases (NOS) on arginine. Cells expressing the enzyme arginosuccinate synthase (ASS-1) and arginosuccinate lyase (ASL) resynthesize arginine from citrulline, to complete the cycle.
